# Draft Genome Sequence of Pseudomonas aeruginosa Strain PA14-UM

**DOI:** 10.1128/MRA.00978-20

**Published:** 2020-11-12

**Authors:** Vincent T. Lee, Reza Ghodssi, Najib M. El-Sayed, Rena D. Malik, Olga G. Goloubeva, Tracy H. Hazen, David A. Rasko

**Affiliations:** a Department of Cell Biology and Molecular Genetics, University of Maryland, College Park, College Park, Maryland, USA; b Department of Electrical and Computer Engineering, Institute for Systems Research, Fischell Department of Bioengineering, Fischell Institute for Biomedical Devices, University of Maryland, College Park, College Park, Maryland, USA; c Center for Bioinformatics and Computational Biology, University of Maryland, College Park, College Park, Maryland, USA; d Department of Surgery, Division of Urology, University of Maryland School of Medicine, Baltimore, Maryland, USA; e Department of Epidemiology & Public Health, University of Maryland School of Medicine, Baltimore, Maryland, USA; f Institute for Genome Sciences, University of Maryland School of Medicine, Baltimore, Maryland, USA; g Department of Microbiology and Immunology, University of Maryland School of Medicine, Baltimore, Maryland, USA; Loyola University Chicago

## Abstract

Pseudomonas aeruginosa is a Gram-negative nosocomial pathogen that is a leading cause of morbidity and mortality in cystic fibrosis patients and immunocompromised individuals worldwide. The isolate examined in this study, PA14-UM, is a well-characterized isolate utilized in studies from the University of Maryland.

## ANNOUNCEMENT

Pseudomonas aeruginosa is an opportunistic pathogen that is associated with significant global morbidity and mortality among hospitalized burn and cystic fibrosis patients, as well as immunocompromised individuals (1). P. aeruginosa PA14, originally isolated by the Ausubel laboratory (2), was acquired by the laboratory of Steven Lory and passed on to the laboratory of Vincent Lee, where it has been named PA14-UM to indicate the version of PA14 that resides at the University of Maryland (3–10). PA14 is a commonly used strain for studies of P. aeruginosa and represents a lineage that is distinct from another commonly used reference isolate, PAO1. Since PA14 is investigated for studies in signal transduction, motility, biofilm formation, and virulence, a comprehensive sequence of strain derivatives will allow comparison of data generated from different labs, such as those completed with PAO1 (11).

The isolate has been stored in the Lee laboratory at −80°C as a 20% glycerol stock. This genome will serve as the reference for this isolate utilized in studies from the University of Maryland, as it has been noted recently that isolates from different stocks of supposedly the same isolate often have genomic and phenotypic differences (11). This isolate was grown for DNA isolation in LB medium overnight at 37°C with agitation, and the purified genomic DNA was collected from 1 ml of culture using a commercially available kit (ArchivePure DNA cell/tissue kit; 5 Prime, Hilden, Germany). Sequencing was performed by the Microbial Genome Sequencing Center (https://www.migscenter.com/). Library prep was conducted using a modified version of the Nextera DNA kit with no size selection, and the library was sequenced on the NextSeq 550 platform (12). A total of 5,943,300 raw read pairs of 150 bp were generated. Raw sequencing reads were filtered to remove contaminating phiX reads using BBDuk of the BBTools software suite (https://sourceforge.net/projects/bbmap/). The raw reads were also filtered to remove contaminating Illumina adapter sequences and quality trimmed using Trimmomatic v0.36 (13). All software was used with default values unless otherwise noted. The resulting filtered reads were assembled using SPAdes v3.14.1 (14). The assemblies were then filtered to contain only contigs longer than 500 bp with a k-mer coverage of ≥5×. The genome sequence consists of 461 contigs with an *N*_50_ value of 26,344 bp and a sequencing coverage of 137.8×. The resulting genome size is 6,467,793 bp with a G+C content of 66.33%. The genome was annotated with PGAP v4.12 (15). Comparison of the PA14-UM isolate to the parental strain, UCBPP_PA14 (GenBank accession number CP000438.1) (16), using nucmer v4.0 (17) identified a total of 283 single nucleotide variants.

This genome sequence serves as the reference for this isolate to be utilized in future studies from the University of Maryland.

### Data availability.

All data have been released, and the accession numbers are as follows. The genome assembly is at GenBank under accession number JACFYT000000000, and the raw reads have been submitted to the SRA under accession number SRR12339385.
